# MicroRNA-21-5p Reduces Hypoxia/Reoxygenation-Induced Neuronal Cell Damage through Negative Regulation of CPEB3

**DOI:** 10.1155/2021/5543212

**Published:** 2021-12-02

**Authors:** Bin Wang, Ping Yu, Wei Lin, Zhaohui Zhai

**Affiliations:** ^1^Department of Neurology, Affiliated Hospital of Weifang Medical College, Weifang, Shandong 261031, China; ^2^Pain Department, Weifang People's Hospital, Weifang, Shandong 261041, China; ^3^Department of Plastic Surgery, Plastic Surgery Hospital of Weifang Medical University, Weifang, Shandong 261042, China

## Abstract

**Objectives:**

To explore the role of microRNA-21-5p (miR-21-5p) in hypoxia/reoxygenation- (H/R-) induced HT22 cell damage.

**Methods:**

The hypoxia/reoxygenation (H/R) model was established in mouse neuronal cells HT22. Cell Counting Kit-8 (CCK-8) and qRT-PCR were used to determine the effects of H/R treatment on cell viability and miR-21-5p expression. HT22 cells were transfected with miR-21-5p mimic or negative control (NC) followed by the induction of H/R; cell viability, apoptosis, and SOD, MDA, and LDH activities were detected. Besides, the apoptosis-related proteins including BAX, BCL2, cleaved caspase-3, and caspase-3 as well as proteins of EGFR/PI3K/AKT signaling pathways were measured by Western blot. To verify the target relation between cytoplasmic polyadenylation element binding protein 3 (CPEB3) and miR-21-5p, luciferase reporter gene experiment was performed. After cotransfection with miR-21-5p mimic and CPEB3 plasmids, the reversal effects of CPEB3 on miR-21-5p in H/R damage were studied.

**Results:**

H/R treatment could significantly reduce the cell viability (*P* < 0.05) and miR-21-5p levels (*P* < 0.05) in HT22 cells. After overexpressing miR-21-5p, cell viability was increased (*P* < 0.05) under H/R treatment, and the apoptosis rate and the levels of apoptosis-related proteins were suppressed (all *P* < 0.05). Furthermore, SOD activity was increased (*P* < 0.05), while MDA and LDH activity was decreased (both *P* < 0.05). Besides, miR-21-5p could restore the activation of the EGFR/PI3K/AKT signaling pathway inhibited by H/R treatment (all *P* < 0.05). The luciferase reporter gene experiment verified that CPEB3 is the target of miR-21-5p (*P* < 0.05). When coexpressing miR-21-5p mimic and CPEB3 in the cells, the protective effects of miR-21-5p under H/R were reversed (all *P* < 0.05), and the activation of the EGFR/PI3K/AKT pathway was also inhibited (all *P* < 0.05).

**Conclusion:**

This study showed that miR-21-5p may regulate the EGFR/PI3K/AKT signaling pathway by targeting CPEB3 to reduce H/R-induced cell damage and apoptosis.

## 1. Introduction

Cerebrovascular disease is the primary cause of human disability. It was characterized by high morbidity, disability, and mortality, which threaten human health seriously [1]. It is known that thrombolytic therapy is beneficial to the recanalization of occluded cerebrovascular and the timely recovery of ischemic brain tissue. With the continuous development of this treatment, the lives of countless ischemic stroke patients have been saved, but it has also caused serious ischemia/reperfusion (I/R) injuries [2]. I/R can cause a variety of serious brain damage, such as cerebral edema, cerebral hemorrhage, neurovascular damage, neuronal necrosis, and inflammation [3]. The pathophysiological mechanism of I/R injury is very complicated, mainly including oxidative stress, calcium overload, cell apoptosis, and inflammatory response [4]. However, as of now, there is no clinically effective drug for the treatment of brain I/R [5]. Therefore, it is of great clinical significance to actively explore and study the pathogenesis of brain I/R injury and find a treatment route.

In recent years, increasing studies have shown that microRNAs (miRNAs) are closely associated with various neurological diseases (including ischemic stroke) [6, 7]. miRNAs are a series of endogenous single-stranded noncoding small RNA molecules discovered in recent years, composed of about 22 nucleotides, and regulate gene expression by targeting the 3′-untranslated regions (UTR) of mRNA [8]. Many diseases are characterized by the abnormal regulation of miRNAs, involving cell functions such as proliferation, apoptosis, and differentiation [9]. Therefore, miRNAs have the potential to treat many diseases and have received extensive attention in recent years [10]. However, the role of miRNAs in ischemic stroke is still unknown. Shi et al. found that miRNA-137 could protect neurons and reduce I/R damage by regulating the Notch signaling pathway [11]. It is known that miR-21-5p is one of the oncogene miRNAs that has been extensively studied. Its main function is to target multiple genes involved in endogenous and exogenous apoptosis pathways, to exert antiapoptotic effects [12]. In the neural system, Yao et al. found that miR-21-5p could improve cerebral ischemic I/R injury and reduce the damage of the blood-brain barrier through the MAPK signaling pathway in a rat neural I/R model [13].

The present study used neuronal cells HT22 to establish a hypoxia/reoxygenation (H/R) model in vitro to simulate neuronal I/R damage, and the protective effects and underlying mechanism of miR-21-5p in H/R injury were studied. We hope to provide valuable clues and theoretical basis for mitigating brain I/R damage.

## 2. Materials and Methods

### 2.1. Experimental Materials

HT22 mouse hippocampal neuron cells were purchased from Shanghai Fuheng Biotechnology Co., Ltd.; Dulbecco's modified Eagle's medium (DMEM), fetal bovine serum, penicillin (100 U/mL)-streptomycin (100 *μ*g/mL) antibiotics, and EDTA-trypsin digestion liquids were purchased from Gibco, USA. The cell hypoxia culture chamber was purchased from Billups-Rothenberg, USA. The construction of miR-21-5p negative control (NC), miR-21-5p mimic, empty plasmid, cytoplasmic polyadenylation element-binding protein 3 (CPEB3) overexpression plasmid, and luciferase control reporter vector was all completed by Shanghai Jikai Gene Chemical Technology Company, and the transfection reagent Lipofectamine 2000 (#11668027) was purchased from the United States Invitrogen Corporation. Cell Counting Kit-8 (CCK-8, #CK04) for cell viability measurement was purchased from Dojindo, Japan. Annexin V-FITC and PI dual staining assay kit (#C1062S) for cell apoptosis was purchased from China Beyotime Biotechnology Co., Ltd. The RNA extraction reagent Trizol (#10296010) was purchased from Invitrogen, USA, and the reverse transcription (#639505) and SYBR fluorescent PCR kits (#RR430A) were purchased from Takara, Japan. RIPA lysis buffer (#P0013B), BCA protein concentration determination kit (#P0012), primary and secondary antibody diluents (#P0256, P0258), blocking solution (#P0252), and ECL luminescence coloring solution (#P0018FM) were all purchased from China Beyotime Biotechnology Co., Ltd., and PVDF membranes (#IEVH00005) were purchased from Millipore, USA. Primary antibodies including B cell lymphoma-2 (BCL2, #3498), BCL2-associated X (BAX, #2772), cleaved caspase-3 (#9661), caspase-3 (#9662), epidermal growth factor receptor (EGFR) (#4267), phosphorylated-protein kinase B (p-AKT, #4058), AKT (#4685), phosphorylated-phosphatidylinositol 3-kinase (p-PI3K, #17366), PI3K (#4292), and *β*-actin (#4970) were purchased from Cell Signaling Technology in the United States. The primary antibody for CPEB3 (ab10883) was purchased from Abcam, USA. Cell superoxide dismutase (SOD, #A001-3-2), malondialdehyde (MDA, #A003-4-1), and lactate dehydrogenase (LDH, #A020-1-2) detection kits were purchased from Nanjing Jiancheng Institute of Bioengineering, China.

### 2.2. Experimental Method

#### 2.2.1. Cell Culture

The HT22 mouse hippocampal neuronal cell line was cultured in DMEM containing 10% fetal bovine serum (FBS) and 1% penicillin (100 U/mL)-streptomycin (100 *μ*g/mL) antibiotics. The medium was cultured in a humidified incubator containing 5% CO_2_ at 37°C. The cells were digested with EDTA-trypsin digestion solution for passage when 90% confluence was reached. All treatments were conducted in cells at passages 4-8.

#### 2.2.2. Establishment of Cell H/R Model

In the H/R experiment, HT22 cells were cultured in a hypoxic chamber with 5% CO_2_ and 95% N_2_ at 37°C for 24 h, during which the cell culture medium was replaced with serum-free and sugar-free DMEM, and then, the cells were returned to a normal humidified incubator cultured at 37°C, with 95% air and 5% CO_2_ (normal oxygen conditions) and continue reoxygenation for 24 h.

#### 2.2.3. Cell Grouping and Transfection

To study the effects of miR-21-5p under H/R treatment, HT22 cells were divided into 4 groups: (1) control group (HT22 cells without any treatment); (2) H/R group (hypoxia 24 h/reoxygenation 24 h); (3) miR-21-5p negative control (NC)+H/R group (cells transfected with miR-21-5p NC followed by H/R treatment); and (4) miR-21-5p mimic+H/R group (cells transfected with miR-21-5p mimic followed by H/R treatment).

To study the association between CPEB3 and miR-21-5p under H/R treatment, HT22 cells were divided into 4 groups: (1) NC+H/R group; (2) miR-21-5p mimic+H/R group; (3) miR-21-5p mimic+CPEB3 plasmid negative control (OE-NC)+H/R group (cells transfected with the miR-21-5p mimic and CPEB3 plasmid NC followed by H/R treatment); and (4) miR-21-5p mimic+CPEB3 overexpression (OE)+H/R group (cells transfected with miR-21-5p mimic and CPEB3 OE plasmid followed by H/R treatment).

According to the instructions, Lipofectamine 2000 was used for cell transfection; about 48 h after transfection, HT22 cells were treated with H/R and followed by experiments.

#### 2.2.4. Cell Viability Determined by Cell Counting Kit-8 (CCK-8)

HT22 cells (1 × 10^4^ cells/well) were inoculated in a 96-well plate and cultured overnight to make the cells adhere to the bottom of the plate. After the corresponding treatments, 10 *μ*L of CCK-8 reagent was added to each well, and cells were then put back into the incubator and continue culturing for 2 h. After that, the 96-well plates were taken out and placed in a multifunctional microplate reader to measure the absorbance (OD) value of each well at 450 nm, and the relative cell viability of HT22 was calculated based on this.

#### 2.2.5. Detection of Apoptosis by Flow Cytometry

Annexin V-FITC and PI double staining was used to detect cell apoptosis. Specifically, HT22 cells (1 × 10^5^ cells/well) in a 6-well culture plate were digested with trypsin digestion solution without EDTA after corresponding treatments. After washing the cells with precooled PBS, cells were then centrifuged at 3000 rpm for 5 min to collect the cell pellet, and 500 *μ*L of binding buffer was then added to resuspend the cells. After that, 10 *μ*L Annexin V-FITC staining solution and 10 *μ*L PI staining solution were added to the cell suspension in sequence according to the instructions. After incubating at room temperature for 15 minutes in the dark, apoptosis was immediately measured with a flow cytometer and analyzed with FlowJo software.

#### 2.2.6. Cell Damages Assessed by Measuring the Level of SOD, MDA, and LDH

The corresponding kits were used to measure SOD activity, MDA, and LDH content of HT22 cells; detections and analysis were carried out according to the kit instructions.

#### 2.2.7. Cell Protein Expression Levels Measured by Western Blotting

The RIPA lysis buffer was used to extract HT22 cell protein, which was then centrifuged at 10,000 rpm for 10 min to extract supernatant; bicinchoninic acid protein concentration determination kit was then used to measure the protein concentration. After that, 20 *μ*g of protein samples was separated on a 10% SDS-PAGE gel and transferred to a PVDF membrane. The PVDF membrane was incubated in blocking buffer for 1 h, and then, anti-BAX, BCL2, cleaved caspase-3, caspase-3, CPEB3, EGFR, p-AKT, AKT, p-PI3K, PI3K, and *β*-actin antibodies (dilution ratio 1 : 2000) were added and further incubated overnight. The next day, the membrane was washed with Tris-buffered saline/Tween 20 and then incubated with the corresponding HRP-conjugated secondary antibody for 1 h. Efficient chemiluminescent (ECL) solution was used to measure the protein expression level in the imaging development system. *β*-Actin was used as an internal reference to calculate the relative expression level of cell proteins.

#### 2.2.8. Cell RNA Expression Measured by Real-Time Fluorescent Quantitative PCR

The total HT22 cell RNA was extracted with Trizol reagent, and the light absorption ratio at 260 nm/280 nm was measured with a multifunctional microplate reader to determine the RNA content. Then, reverse transcription of RNA into cDNA was carried out with a reverse transcription kit, and SYBR green PCR amplification kit was used for DNA amplification. The primer sequence was as follows: U6: upstream primer 5′-CTCGCTTCGGCAGCACA-3′, downstream primer 5′-AACGCTTCACGAATTTGCGT-3′; miR-21-5p: upstream primer 5′-CTCGCTTCGGCAGCACA-3′, downstream primer 5′-AACGCTTCACGAATTTGCGT-3′; CPEB3: upstream primer CATTATGGCACTTAACACCAGGA, downstream primer AGAAGGCGTCTTCAAAGGGAA; and *β*-actin: upstream primer GTGACGTTGACATCCGTAAAGA, downstream primer GCCGGACTCATCGTACTCC. The expression level of miR-21-5p and CPEB3 genes was determined by using U6 and *β*-actin as internal controls, respectively, and the relative expression levels of target genes were calculated using the 2^-△△Ct^ method.

#### 2.2.9. Verification of Target Gene by Luciferase Reporter Gene

The TargetScan software was used to predict the potential targets of miR-21-5p. To construct a reporter vector containing the miR-21-5p target, a gene fragment containing the 3′-UTR of the predicted target (CPEB3) was synthesized and then cloned into the pGL3 luciferase control reporter vector. The recombinant plasmids were named pGL3-CPEB3-wild type (WT) and pGL3-CPEB3-mutant type (MUT). To evaluate the effect of miR-21-5p binding on luciferase expression, we cotransfected miR-21-5p mimic or control into HEK293 cells, and 48 h after transfection, the luciferase activity was measured by the dual-luciferase reporter system (Promega), and the firefly luciferase activity was standardized to the expression of renin luciferase.

### 2.3. Statistical Analysis

All experiments were repeated 3 times independently. The measurement data generated in the experiment are all represented with the mean ± standard deviation. SPSS 20.0 software was used for analysis; the comparison between different groups was performed by Student's *t*-test. *P* < 0.05 was considered as significant difference.

## 3. Results

### 3.1. Expression of miR-21-5p and Changes in Cell Viability under H/R Conditions

**Figure 1:**
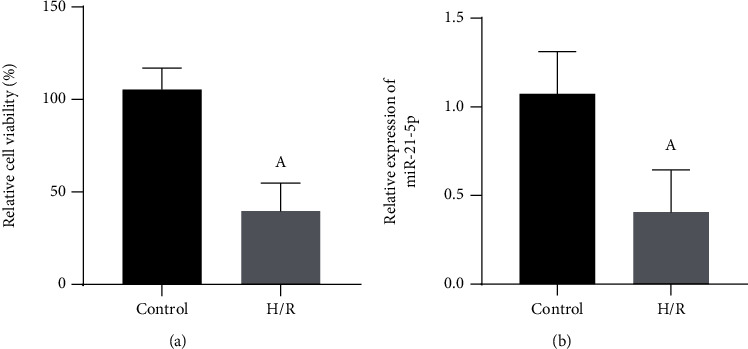
The effect of H/R induction on the viability of HT22 cells and the expression of miR-21-5p (*n* = 3). (a) Changes in the viability of HT22 cells under H/R conditions. (b) Changes in the expression of miR-21-5p in HT22 cells under H/R conditions. Note: Student's *t*-test was used to compare the H/R group with the control group, ^a^*P* < 0.05.

As shown in Figure 1(a), HT22 cells exposed to hypoxia for 24 h and then reoxygenated for 24 h can significantly reduce the cell viability (*P* < 0.05). Besides, as shown in Figure 1(b), the qRT-PCR experiment showed that compared with the control group (control), H/R can significantly reduce the expression of miR-21-5p in HT22 cells (*P* < 0.05).

### 3.2. miR-21-5p Overexpression Inhibits H/R-Induced HT22 Cell Damage

**Figure 2:**
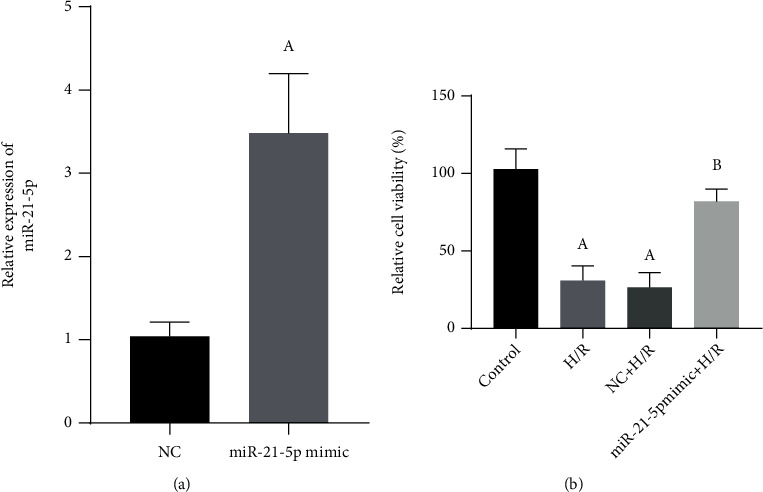
The effect of miR-21-5p overexpression on H/R-induced cell damage (*n* = 3). (a) Verification of the overexpression of miR-21-5p in cells transfected with miR-21-5p mimic. (b) The effects of overexpression of miR-21-5p on cell viability in HT22 cells under H/R conditions. Note: Student's *t*-test was used. Compared with the control group, ^a^*P* < 0.05; compared with the H/R group, ^b^*P* < 0.05.

To further study the function of miR-21-5p in H/R-treated HT22 cells, as shown in Figure 2(a), we first transfected miR-21-5p mimic to overexpress miR-21-5p in HT22 cells (*P* < 0.05); as shown in Figure 2(b), the CCK-8 method results confirmed that the overexpression of miR-21-5p reversed the decreases of cell viability after H/R treatment (*P* < 0.05). Also, as shown in Table 1, H/R can induce a decrease in the SOD activity in HT22 cells (*P* < 0.05), while the activities of cell damage products including MDA and LDH were increased (both *P* < 0.05), which however can be significantly reversed when miR-21-5p was overexpressed (all *P* < 0.05).

### 3.3. miR-21-5p Overexpression Inhibits H/R-Induced HT22 Cell Apoptosis

**Figure 3:**
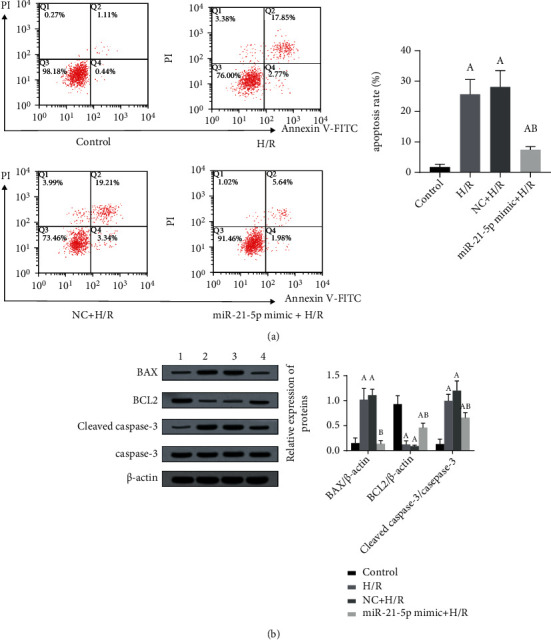
The effect of miR-21-5p overexpression on H/R-induced apoptosis (*n* = 3). (a) Under H/R conditions, the effect of overexpression of miR-21-5p in HT22 cells on cell apoptosis; (b) under H/R conditions, changes in the expression of apoptosis-related proteins in cells overexpressing miR-21-5p. The experimental groups are as follows: 1: control group; 2: H/R group; 3: NC+H/R group; 4: miR-21-5p mimic+H/R group. Note: Student's *t*-test was used. Compared with the control group, ^a^*P* < 0.05; compared with the H/R group, ^b^*P* < 0.05.

As shown in Figure 3(a), H/R treatment can significantly induce apoptosis in HT22 cells (*P* < 0.05), and when cells overexpress miR-21-5p, H/R-induced apoptosis can be significantly inhibited (*P* < 0.05), and as shown in Figure 3(b), the changes in H/R-induced apoptosis-related proteins were also reversed by the miR-21-5p overexpression (all *P* < 0.05).

### 3.4. Overexpression of miR-21-5p Restores the EGFR/PI3K/AKT Signaling Pathway Inhibited by H/R

**Figure 4:**
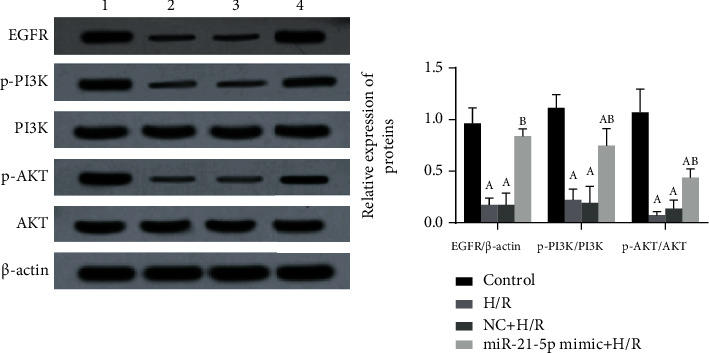
The effects of miR-21-5p overexpression on the activation of the EGFR/PI3K/AKT signaling pathway (*n* = 3). The experimental groups are as follows: 1: control group; 2: H/R group; 3: NC+H/R group; 4: miR-21-5p mimic+H/R group. Note: Student's *t*-test was used. Compared with the control group, ^a^*P* < 0.05; compared with the H/R group, ^b^*P* < 0.05.

To clarify the mechanism of miR-21-5p's protective effect, we also measured the expression changes of proteins involved in the EGFR/AKT/PI3K signaling pathway. As shown in Figure 4, H/R injury significantly inhibited the activation of the EGFR/AKT/PI3K signaling pathway (all *P* < 0.05); however, when miR-21-5p was overexpressed in HT22 cells, the expression of EGFR protein was significantly increased (*P* < 0.05), and the AKT and PI3K proteins also had different degrees of phosphorylation at the same time (all *P* < 0.05).

### 3.5. CPEB3 Is the Target of miR-21-5p

**Figure 5:**
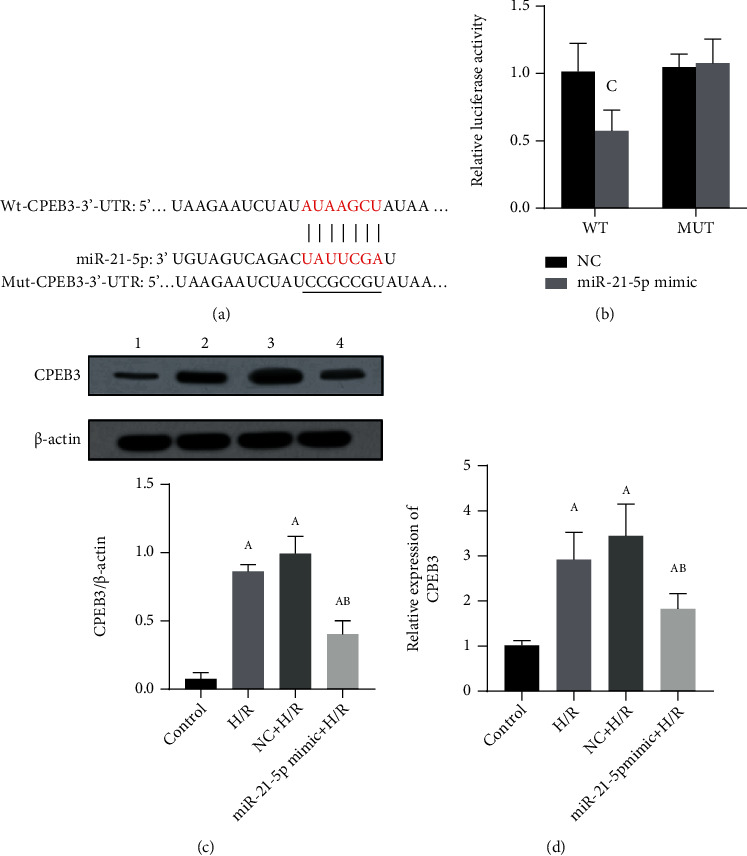
The verification of CPEB3 as a target of miR-21-5p (*n* = 3). (a) CPEB3 as the target of miR-21-5p. (b) Luciferase reporter gene verification. (c) Protein expression level of CPEB3 when miR-21-5p was overexpressed in HT22 cells. (d) The expression level of CPEB3 mRNA when miR-21-5p was overexpressed in HT22 cells. The experimental groups are as follows: 1: control group; 2: H/R group; 3: NC+H/R group; 4: miR-21-5p mimic+H/R group. Note: Student's *t*-test was used. Compared with the control group, ^a^*P* < 0.05; compared with the H/R group, ^b^*P* < 0.05; compared with the NC group, ^c^*P* < 0.05.

To further explore the mechanism of miR-21-5p, we also used TargetScan software to predict the potential targets of miR-21-5p. Among the candidate genes, CPEB3 contains miR-21-5p binding sites in its 3′-UTR, as shown in Figure 5(a). Therefore, we established the wild-type (CPEB3-WT) and mutant (CPEB3-MUT) luciferase report vector of CPEB3 RNA. As shown in Figure 5(b), overexpression of miR-21-5p significantly inhibited CPEB3-WT reporter activity (*P* < 0.05) but had no significant effect on CPEB3-MUT reporter activity (*P* > 0.05). Furthermore, as shown in Figures 5(c) and 5(d), when HT22 cells overexpressed miR-21-5p, the mRNA and protein expression of CPEB3 was significantly downregulated (all *P* < 0.05). The above results indicate that the expression of CPEB3 can be negatively affected by miR-21-5p.

### 3.6. Overexpression of CPEB3 Can Reverse the Protective Effect of miR-21-5p in H/R-Induced HT22 Cells

**Figure 6:**
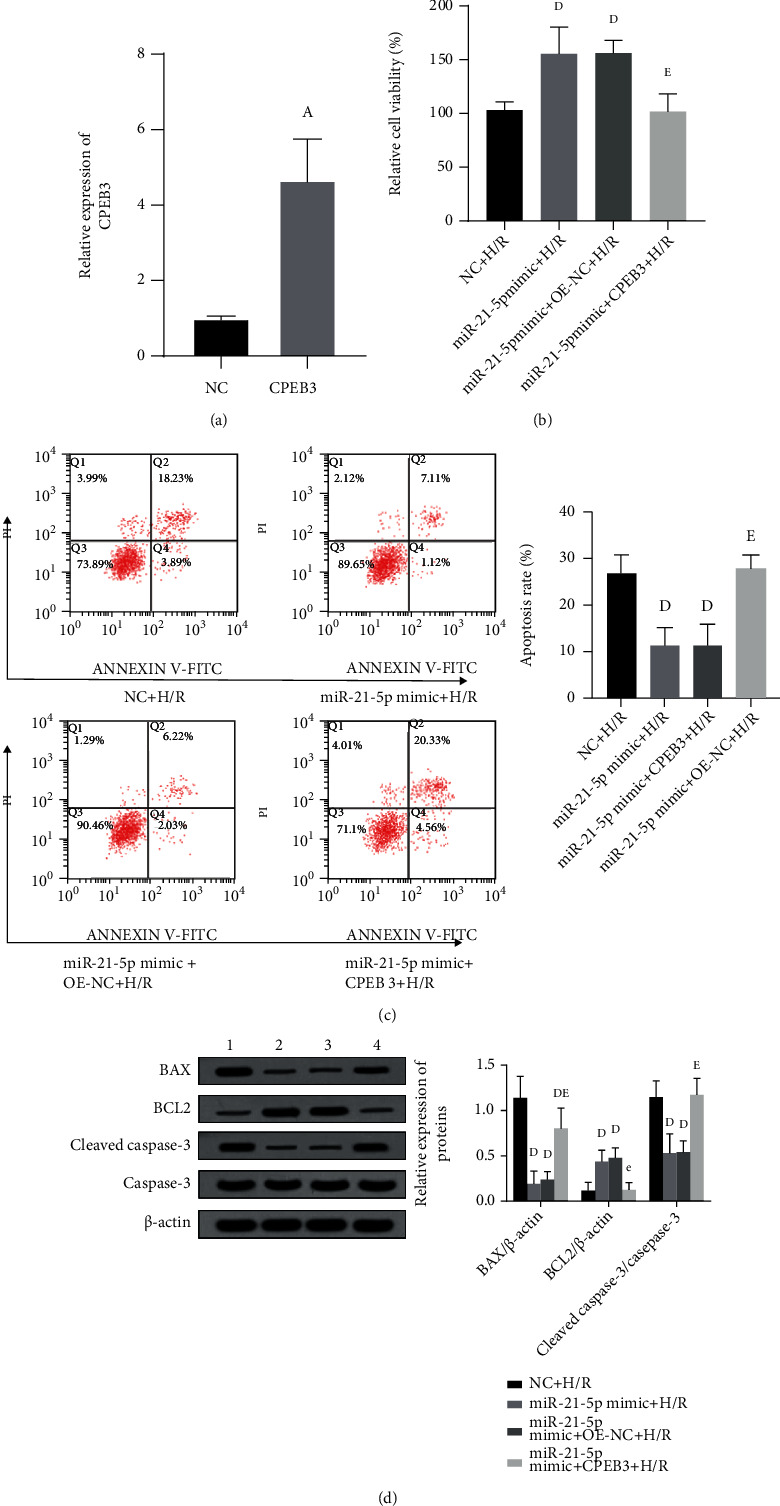
The effect of simultaneous overexpression of miR-21-5p and CPEB3 on H/R-induced cell damage (*n* = 3). (a) The effect of simultaneous overexpression of miR-21-5p and CPEB3 on H/R-induced cell viability. (b) The effect of cotransfection of cells with miR-21-5p mimic and CPEB3 on H/R-induced apoptosis. (c) The effect of simultaneous overexpression of miR-21-5p and CPEB3 on the expression of apoptosis-related proteins in H/R-induced cell. The experimental groups are as follows: 1: NC+H/R group; 2: miR-21-5p mimic+H/R group; 3: miR-21-5p mimic+OE-NC+H/R group; 4: miR-21-5p mimic+CPEB3+H/R group. Note: Student's *t*-test was used. Compared with the NC+H/R group, ^d^*P* < 0.05; compared with the miR-21-5p mimic+H/R group, ^e^*P* < 0.05.

Based on the previous results, we then verified whether CPEB3 can reverse the effect of miR-21-5p. Specifically, HT22 cells were cotransfected with miR-21-5p mimic and CPEB3 overexpression plasmid. As shown in Figure 6(a), CCK-8 results showed that in H/R-treated HT22 cells, overexpression of CPEB3 reversed the protective effect of miR-21-5p on cell viability (*P* < 0.05). Besides, as shown in Table 2, CPEB3 overexpression also inhibited the increase in SOD activity (*P* < 0.05) and the decrease in MDA and LDH content (all *P* < 0.05) caused by miR-21-5p overexpression. Similar reversal effects were also observed in the cell apoptosis, as shown in Figure 6(b). After HT22 cells were treated with H/R, the decrease in apoptosis induced by overexpression of miR-21-5p was also reversed by overexpression of CPEB3 (*P* < 0.05), and as shown in Figure 6(c), the expression of apoptosis-related proteins also showed corresponding changes (all *P* < 0.05).

### 3.7. Overexpression of CPEB3 Can Inhibit the Activation of the EGFR/PI3K/AKT Signaling Pathway Caused by miR-21-5p

**Figure 7:**
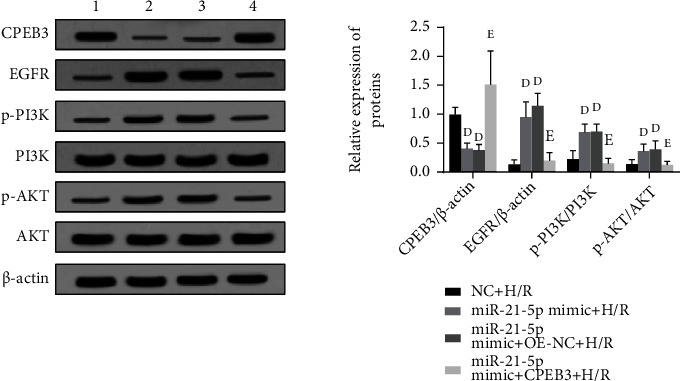
The effects of overexpressing miR-21-5p and CPEB3 simultaneously on the activation of the EGFR/PI3K/AKT signaling pathway (*n* = 3). The experimental groups are as follows: 1: NC+H/R group; 2: miR-21-5p mimic+H/R group; 3: miR-21-5p mimic+OE-NC+H/R group; 4: miR-21-5p mimic+CPEB3+H/R group. Note: Student's *t*-test was used. Compared with the NC+H/R group, ^d^*P* < 0.05; compared with the miR-21-5p mimic+H/R group, ^e^*P* < 0.05.

Previous studies have found that miR-21-5p may play the anti-H/R damage effect by activating the EGFR/PI3K/AKT signaling pathway. As shown in Figure 7, when we cotransfected HT22 cells with miR-21-5p mimic and CPEB3 under the H/R condition, we found that overexpression of CPEB3 can again inhibit the activation of the EGFR/PI3K/AKT signaling pathway, namely, decrease the protein expression level of EGFR (*P* < 0.05) as well as the phosphorylation level of PI3K and AKT proteins to varying degrees (all *P* < 0.05). This result suggests that the miR-21-5p/CPEB3/EGFR/PI3K/AKT signal axis plays a role in regulating H/R injury.

## 4. Discussion

Cerebrovascular disease is one of the three major risk factors that endanger human health, especially cerebral ischemia characterized by hippocampal neuron damage. H/R injury is the main event in the pathogenesis of neurodegeneration, and its main feature is the damage and death of neuronal cells [14]. Based on that, it is imperative to find new therapeutic targets for cerebrovascular disease and to prevent hypoxic-ischemic injury and improve the prognosis of cerebrovascular disease [15]. In this study, mouse neuronal cells HT22 were treated with hypoxia and reoxygenation to establish the H/R cell model, and we found that the H/R could induce neuronal cell damage which was accompanied by the decreased level of miR-21-5p. To further explore the effects of miR-21-5p on HT22 cells, we also transfected miR-21-5p mimic into HT22 cells. The results showed that overexpression of miR-21-5p could restore the activation of the EGFR/PI3K/AKT signaling pathway inhibited by H/R treatment, thereby reducing the H/R-induced cell injury. Besides, we also predicted and verified that CPEB3 may be the target of miR-21-5p.

In recent years, studies have found that miRNAs may be closely associated with the development of cerebrovascular diseases [11–13]. miR-21-5p has also been found to protect different organs and reduce the damage of I/R to tissues [16–18]. As mentioned above, miR-21-5p could reduce I/R-induced neural injury in rats [13], but its underlying mechanism still needs to be clarified. The present study confirmed the protective effects of miR-21-5p in mouse neuronal HT22 cells injured by H/R, such as inhibiting cell apoptosis. We measured the expression levels of apoptosis-related proteins such as BAX, BCL2, cleaved caspase-3, and caspase-3, which are the key proteins that initiate, execute, and regulate the process of apoptosis [19]. We found that when HT22 undergoes apoptosis, the expression of BCL2 decreases, while the expression of BAX and cleaved caspase-3 increases. However, when the cells are overexpressed with miR-21-5p mimic, the expression of the above proteins was inversed, which is consistent with the results of apoptosis measured by flow cytometry, suggesting that miR-21-5p can play an important antiapoptosis role in H/R-induced cells.

Evidence has shown that oxidative stress in the state of cerebral ischemia and reperfusion may be one of the main mechanisms that lead to irreversible brain damage [20]. Specifically, oxidative stress can cause neuronal apoptosis, degeneration, or necrosis, which ultimately leads to brain dysfunction. During the occurrence and development of oxidative stress, SOD is an important free radical scavenger in the body, which can protect brain tissue from free radical damage [21], while MDA is a lipid peroxidation product induced by oxygen free radicals, which can indirectly reflect the degree of cell damage [22]. In addition, LDH leakage, as a marker of cell damage, is considered to be a sensitive biochemical indicator reflecting the level of cell viability and function [23]. Our results showed that after H/R-induced injury in HT22 cells that overexpressed miR-21-5p, the activity of SOD was upregulated, while the contents of MDA and LDH were decreased, suggesting that miR-21-5p has the protective effects on H/R-induced oxidative stress.

**Figure 8:**
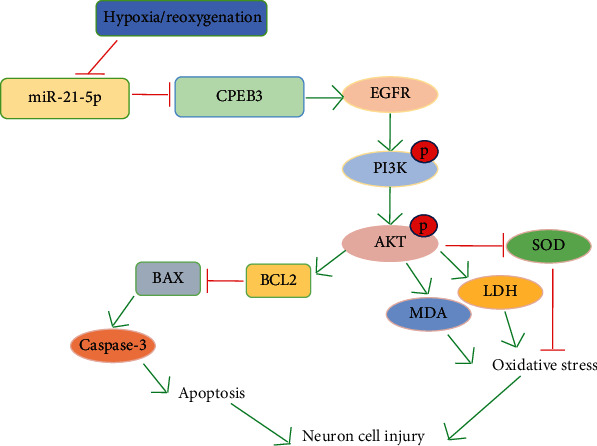
Diagram showing the roles of miR-21-5p/CPEB3 in H/R-induced HT22 cell damage.

In the study of the molecular mechanism, EGFR is known to be widely expressed in the central nervous system. When central neurons are damaged, EGFR is upregulated and activated. Because phosphorylated EGFR can activate mTOR, EGFR may participate in axon regeneration through the PI3K/AKT pathway and promote the recovery of nerve injury [24]. In addition, EGFR can activate a series of downstream signaling pathways, among which the PI3K/AKT signaling pathway is one of the most classical ways of activation. It is known that PI3K is the main downstream effector of receptor tyrosine kinases and G protein-coupled receptors, and it can produce activated serine/threonine-protein kinase AKT and its downstream phospholipids. It transmits the signals of growth factors and cytokines to intracellular messengers, thereby exerting a series of functions in the regulation of cell growth [25]. In this study, we found that overexpression of miR-21-5p restored the activation of the EGFR/PI3K/AKT signaling pathway inhibited by H/R treatment, suggesting that the protective effects of miR-21-5p may be achieved by regulating this pathway. Besides, by using TargetScan software to predict the potential targets of miR-21-5p, we found that the CPEB3 gene may be its target and the luciferase reporter gene experiment was used for the verification. CPEB3, as a member of the CPEBs family, regulates protein translation by regulating cytoplasmic polyadenylation. Abnormal expression of CPEB3 has been found in a variety of cancers, such as cervical cancer and human hepatocellular carcinoma [26]; studies have found that miRNAs can directly target the CPEB3/EGFR axis to regulate tumor progression, but few studies are focusing on the nervous system. In this study, HT22 cells were cotransfected with miR-21-5p mimic and CPEB3 overexpression plasmid. We found that the overexpression of CPEB3 significantly reversed the protective effect of miR-21-5p on H/R-induced cell injury. CPEB3 overexpression promoted H/R-induced cell damage, induced cell apoptosis, and inhibited the activation of the EGFR/PI3K/AKT signaling pathway. These results confirmed that CPEB3 is the target of miR-21-5p and the effects of miR-21-5p on the regulation of the EGFR/PI3K/AKT signaling axis.  Figure 8 shows the roles of miR-21-5p/CPEB3 in H/R-induced HT22 cell damage.

## 5. Conclusion

In summary, this study shows that miR-21-5p may reduce H/R-induced cell damage and apoptosis by targeting CPEB3 and activating the EGFR/PI3K/AKT signaling pathway.

## Figures and Tables

**Table 1:** The effects of miR-21-5p on SOD, MDA, and LDH activity of H/R-induced cells (*n* = 3, mean ± SD).

Indicators	Control	H/R	NC+H/R	miR-21-5p mimic+H/R
SOD (U/mg protein)	153.33 ± 45.09	40.00 ± 26.46^a^	38.00 ± 15.87^a^	105.00 + 47.70^b^
MDA (nmol/mg protein)	1.07 ± 0.40	2.80 ± 0.82^a^	2.33 ± 0.91^a^	1.47 ± 0.55^b^
LDH (U/mg protein)	5.33 ± 1.53	15.67 ± 5.03^a^	17.00 ± 5.29^a^	8.33 ± 2.08^b^

Note: Student's *t*-test was used. Compared with the control group, ^a^*P* < 0.05; compared with the H/R group, ^b^*P* < 0.05.

**Table 2:** The effects of miR-21-5p and CPEB3 on SOD, MDA, and LDH activity in H/R-induced cells (*n* = 3, mean ± SD).

Indicators	NC+H/R	miR-21-5p mimic+H/R	miR-21-5p mimic+OE-NC+H/R	miR-21-5p mimic+CPEB3+H/R
SOD (U/mg protein)	48.33 ± 11.50	121.33 ± 32.02^d^	110.33 ± 19.50^d^	50.67 ± 11.02^e^
MDA (nmol/mg protein)	2.33 ± 0.91	1.47 ± 0.55^d^	1.17 ± 0.60^d^	3.13 ± 1.44^e^
LDH (U/mg protein)	17.67 ± 5.03	7.67 ± 3.21^d^	7.67 ± 3.06^d^	21.00 ± 8.19^e^

Note: Student's *t*-test was used. Compared with the NC+H/R group, ^d^*P* < 0.05; compared with the miR-21-5p mimic+H/R group, ^e^*P* < 0.05.

## Data Availability

The data used to support the findings of this study are included within the article.
